# Mechanisms of escape phenomenon of spinal cord and brainstem in human rabies

**DOI:** 10.1186/1471-2334-5-104

**Published:** 2005-11-16

**Authors:** Sasiwimol Juntrakul, Preecha Ruangvejvorachai, Shanop Shuangshoti, Supaporn Wacharapluesadee, Thiravat Hemachudha

**Affiliations:** 1 Molecular Biology Laboratory for Neurological Diseases, Department of Medicine, Chulalongkorn University Hospital, Rama 4 Road, Bangkok, Thailand; 2 Department of Pathology, Chulalongkorn University Hosital, Rama 4 Road, Bangkok, Thailand

## Abstract

**Background:**

Rabies virus preferentially involves brainstem, thalamus and spinal cord in human furious and paralytic rabies beginning in the early stage of illness. Nevertheless, rabies patient remains alert until the pre-terminal phase. Weakness of extremities develops only when furious rabies patient becomes comatose; whereas peripheral nerve dysfunction is responsible for weakness in paralytic rabies.

**Methods:**

Evidence of apoptosis and mitochondrial outer membrane permeabilization in brain and spinal cord of 10 rabies patients was examined and these findings were correlated with the presence of rabies virus antigen.

**Results:**

Although apoptosis was evident in most of the regions, cytochrome c leakage was relatively absent in spinal cord of nearly all patients despite the abundant presence of rabies virus antigen. Such finding was also noted in brainstem of 5 patients.

**Conclusion:**

Cell death in human rabies may be delayed in spinal cord and the reticular activating system, such as brainstem, thus explaining absence of weakness due to spinal cord dysfunction and preservation of consciousness.

## Background

Clinical presentations of rabies in humans can be categorized as classic (furious and paralytic) and non-classic rabies [1,2]. The latter is almost always associated with bat and some dog variants whereas the classic forms are associated with dog variants. There is no specific genetic pattern of rabies virus associated with either furious or paralytic forms based on an analysis of glyco-, phospho-, nucleoprotein genes [3]. Analysis of regional distribution of rabies virus antigen in the central nervous system (CNS) revealed similar pattern [4]. Rabies virus antigen preferentially localizes in the spinal cord and brainstem and thalamus, basal ganglia if the survival period is 7 days or less regardless of the clinical forms [4]. Such brainstem and thalamus predilection is also evident in animals [5].

Serial electrophysiologic studies of peripheral nerve in furious rabies patients revealed a sub-clinical evidence of anterior horn cell dysfunction in the spinal cord [6,7]. These patients did not exhibit any demonstrable weakness of the arms and legs. It is only at the time when furious rabies patients become comatose that weakness of all limb musculatures can be demonstrated. In paralytic rabies patients, limb weakness is explained by peripheral nerve and not the anterior horn cell dysfunction [6,7]. This is also confirmed by prominent inflammation and demyelination in the peripheral nerve of these paralytic rabies patients [6-10].

These findings raised important questions why clinical weakness due to spinal cord dysfunction does not develop in rabies patients. Along with this "escape phenomenon" of rabies virus infected spinal cord, it is also intrigued that rabies patients do not have depressed consciousness during the most entire clinical course despite an enormous amount of rabies virus since the early stage in the brainstem and thalamus, structures which are crucial in maintaining alertness and form an integral part of reticular activating system.

Spinal cord motoneuron resists to cytolysis and apoptosis in spinal cord and anterior horn cell culture system with rabies virus infection [11]. In vivo, despite the massive infection of the spinal cord in infected rat neonates, only a few motoneurons were apoptotic. Moreover, axon of rabies infected motoneuron was able to elongate at a comparable rate in virus-infected and noninfected cultures indicating that metabolic activity was maintained in these infected cells. In contrast, a large proportion of hippocampus neurons were apoptotic shortly after infection. These suggest that spinal cord motoneurons survive rabies virus infection because the viral induction of apoptosis is delayed in these neurons.

Apoptosis or programmed cell death can be induced by multiple insults which proceeds through the mitochondrial pathway – mitochondrial outer membrane permeabilization (MOMP) in which cytochrome c appears to be a major inducer of the entire cascade though the activation of caspase-9 and -3 [12].

The objective of our study was to determine whether brainstem and spinal cord in rabies patients are lacking of either apoptosis or mitochondrial outer membrane permeabilization (MOMP) or both which, in turn, may explain this escape phenomenon. We also compared the degree of rabies virus infection and apoptosis and MOMP at various CNS regions of furious and paralytic rabies patients.

## Materials and methods

### Materials

Formalin fixed paraffin embedded CNS tissue of 10 rabies patients processed between 1987 and 2005 were included in this study. Five rabies patients presented as furious and the remaining had paralysis. Characteristics of these patients were summarized in Table 1. All patients did not receive any intensive care support and had evidence of hypoxia and cardiovascular collapse during the last 24–36 hours before death. Post-mortem examinations were performed within 24 h of death. Brain and spinal cord were fixed in formalin for 7 days. Sections of frontal, temporal, hippocampus, parietal, occipital, thalamus, basal ganglia, cerebellum, midbrain, pons, medulla, cervical, thoracic, lumbosacral enlargement were subsequently embedded in paraffin, sectioned and examined for the presence of rabies virus antigen and apoptosis and MOMP.

**Table 1:** Characteristics of patients with rabies.

Patient No.	Age (yrs), Sex	Incubation time and site of bite	Survival period (days)*	History of Immunization
Furious				
H1	9, male	1 month; dog bite on right buttock	5	-
H3	22, female	3 months; dog bite on right ankle	8	-
H5	55, female	2 months; dog bite on right hand, left leg, left breast	4	-
H6	14, male	1 month; dog bite on buttock	5	-
H8	31, male	3 months before; dog bite on right foot	5	-

Paralysis				
H2	15, female	3 months; dog bite on finger	16	-
H4	81, male	2 months; dog bite on left calf	7	+
H7	61, male	4 months; dog bite on right leg	13	-
H9	43, female	3 months before; dog bite on left hand	9	-
H10	18, male	uncertain; dog bite on right leg	13	-

* = interval between onset and death

### Methods

#### 1. Slide preparation

Three μm-thick paraffin sections of formalin-fixed tissue were mounted on silane-coated slides [2% 3-aminopropyltriethoxysilane (Sigma, USA)].

#### 2. Immunoperoxidase staining for rabies antigen and cytochrome c

Sections were stained by DAKO EnVision™ System kit, HRP (DAKO Corporation, CA, USA) after deparaffinized by xylene and ethanol and then antigen was retrieved by pressure cooker with citrate buffer for 1 min. Briefly, sections were incubated for 10 min with 3%H_2_O_2 _to eliminate endogenous peroxidase, washed in phosphate-buffered saline (PBS), incubated for 20 min with 3% horse serum to block nonspecific staining, and incubated for 60 min with anti-rabies nucleocapsid polyclonal antibody (Bio-rad, France) at a dilution of 1:80 or anti-cytochrome c monoclonal antibody (Santa Cruz Biotechnology, USA) at a dilution of 1: 4000. After two 3-min rinses in PBS, sections were incubated in DAKO EnVision™ System kit, HRP reagent (DAKO Corporation, CA, USA) as secondary antibody for 30 min. Slides were washed with PBS again and incubated for 10 min with peroxidase substrate [diaminobenzidine (DAB; Sigma, USA) 0.5 mg/ml and 30%H_2_O_2 _in Tris-HCl buffer with 1 M Imidazole]. After rinsed by tap water, the stain was couterstained with hematoxylin.

#### 3. Detection of apoptosis

To evaluate whether cell death was due to apoptosis, we used the ApopTag^® ^Plus Peroxidase *In Situ *Apoptosis Kit (Intergen Company, USA) as well as TUNEL assay (Terminal deoxynucleotidyl transferase (TdT)-mediated dUTP-digoxigenin nick end labelling assay) for detection. The kit detects apoptotic cells by peroxidase staining detection of the digoxigenin-labeled 3'-OH DNA ends generated by DNA fragmentation, and typically localized in morphologically identifiable nuclei and apoptotic bodies.

Sections were stained by ApopTag^® ^Peroxidase kit after deparaffinized by xylene and ethanol and then permeabilized cell by proteinase K. Briefly, sections were incubated for 5 min with 3%H_2_O_2 _to quench endogenous peroxidase and apply equilibration buffer. Sections were then incubated in a humidified chamber at 37°C for 1 hour with working strength TdT enzyme (TdT enzyme mediated digoxigenin-dUTP), kept in a coupling jar containing working strength stop/wash buffer, and incubated for 10 min at room temperature. Slides were washed with PBS, incubated with anti-digoxigenin peroxidase conjugate in humidified chamber for 30 min at room temp and washed again. Developing color was done with peroxidase substrate as previously described. Finally, slides were washed in water and couterstained with hematoxylin.

##### Quantitation and controls

In order to clarify how cells were shown to be neuronal and not another cell types. The specimens were serially sectioned and stained as follows: hematoxylin and eosin (H&E), rabies antigen, cytochrome c, TUNEL and neurofilament protein (as a neuronal marker) (DAKO Corporation, CA, USA). Number or density of neurons in each slide was examined by H&E and was found to be correlated with neurofilament immunostaining technique.

The number of rabies antigen-positive cells and cells with MOMP and TUNEL positive cells in various areas was graded on a 0 – 4 scale from none to most abundant [4] by 3 readers (SJ, SS and TH) independently and where disagreement occurred the respective cases were re-examined and a consensus reached. Scale measurements of 0 – 4 were based on the followings: (0)-no antigen positive neuron in all fields; (1) 1 – 25 % antigen positive neuron(s) in the whole section; (2) 26 – 50% antigen positive neurons in the whole section; (3) 51 – 75% antigen positive neurons in the whole section; (4) 76 – 100 % antigen positive neurons in the whole section.

Brain sections from a patient with lung cancer with no CNS complications served as negative controls.

## Results

### Regional CNS distribution of rabies virus antigen and apoptosis

#### Rabies virus antigen

**Figure 1:**
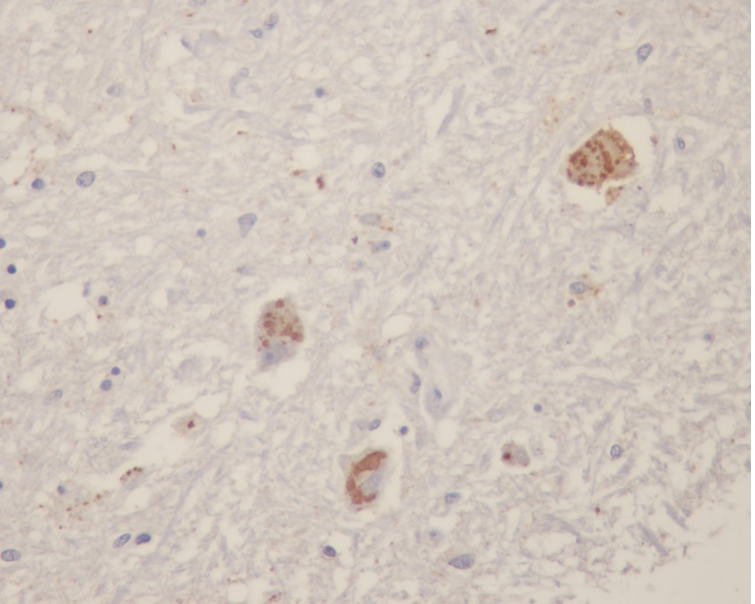
Rabies antigen positive neurons in brainstem at midbrain region of a paralytic rabies patient (no. H4 – see text and Table 1).

**Figure 2:**
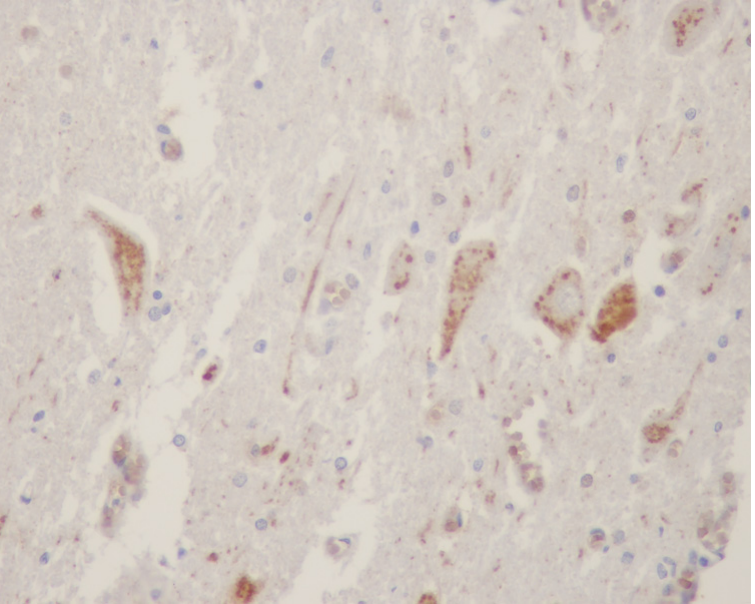
Rabies antigen positive neurons in spinal cord at thoracic region of a furious rabies patient (no. H3 – see text and Table 1).

The overall regional distribution of rabies viral antigen was roughly similar in terms of number and location to that previous report [4]. Rabies antigen-containing neurons were found predominantly in the brain stem and spinal cord, dorsal and ventral horn neurons, thalamus and basal ganglia particularly in patients who had survival periods of 7 days or less regardless of clinical forms (Patient nos. 1, 5, 6 and 8 in furious and 4 in paralytic group) (Table 2) (Figure 1). Those who died later than 7 days (Patient nos.3 in furious and 2, 7, 9, and 10 in paralytic group) had rabies viral antigen disseminated throughout the whole neuraxis (Table 3) (Figure 2).

**Table 2:** Distribution of rabies virus and cytochrome c in CNS of human rabies patients who survived 7 days or less. (Numbers in bold and italic designated discrepancy between rabies antigen positive- and cytochrome c positive neurons in particular region).

**Patient No.**	**Survival period**	**Antigen**	**Frontal**	**Temporal**	**Hippocampus**	**Parietal**	**Occipital**	**Thalamus**	**Basal-ganglia**	**Cerebellum**	**Midbrain**	**Pons**	**Medulla**	**Cervical**	**Thoracic**	**Lumbar**	**Sacrum**
**Furious**																	
H1	5	Rabies	0	0	0	0	0	3	2	1	***3***	***3***	***3***	***3***	***4***	***4***	nd**
		Cyto C*	0	0	1	0	0	3	1	4	***0***	***0***	***0***	***0***	***1***	***0***	nd
H5	4	Rabies	2	2	2	3	2	3	3	3	4	4	4	***3***	***3***	***4***	***4***
		Cyto C	2	1	4	3	4	4	4	2	4	4	4	***0***	***1***	***2***	***2***
H6	5	Rabies	2	0	1	3	0	2	3	2	3	3	3	3	***3***	***3***	***3***
		Cyto C	1	3	2	4	4	4	4	3	4	4	4	2	***0***	***0***	***0***
H8	5	Rabies	1	3	4	3	2	4	4	2	3	4	4	nd	nd	nd	nd
		Cyto C	1	4	4	4	4	4	3	2	3	4	4	nd	nd	nd	nd
**Paralysis**																	
H4	7	Rabies	2	1	2	2	2	3	2	2	4	4	3	***3***	***3***	3	nd
		Cyto C	2	0	2	2	3	0	3	2	4	4	3	***1***	***1***	nd	nd

* = cytochrome c ** = not done (sample not available)

**Table 3:** Distribution of rabies virus and cytochrome c in CNS of human rabies patients who survived longer than 7 days (Numbers in bold and italic designated discrepancy between rabies antigen positive- and cytochrome c positive neurons in particular region).

**Patient No.**	**Survival period**	**Antigen**	**Frontal**	**Temporal**	**Hippocampus**	**Parietal**	**Occipital**	**Thalamus**	**Basal-ganglia**	**Cerebellum**	**Midbrain**	**Pons**	**Medulla**	**Cervical**	**Thoracic**	**Lumbar**	**Sacrum**
**Furious**																	
H3	8	Rabies	2	3	3	4	3	3	4	2	4	4	4	***4***	***4***	***4***	4
		Cyto C*	0	3	4	0	2	4	4	1	4	4	4	***2***	***1***	***2***	3
**Paralysis**																	
H2	16	Rabies	4	4	4	4	4	4	4	4	***4***	***4***	***4***	***4***	***4***	***4***	nd**
		Cyto C	0	0	0	0	1	3	3	1	***1***	***1***	***0***	***0***	***0***	***0***	nd
H7	13	Rabies	4	3	2	3	3	4	nd	3	4	***4***	4	***4***	***3***	4	***4***
		Cyto C	2	3	nd	4	3	3	nd	1	4	***2***	4	***0***	***0***	nd	***1***
H9	9	Rabies	4	2	nd	4	4	nd	4	4	***4***	nd	***3***	nd	nd	nd	nd
		Cyto C	1	0	nd	2	0	nd	0	3	***1***	nd	***1***	nd	nd	nd	nd
H10	13	Rabies	4	4	4	3	3	4	4	4	***4***	4	4	4	***4***	***4***	***4***
		Cyto C	3	3	1	2	2	4	4	4	***1***	4	4	4	***3***	***1***	***0***

*= cytochrome c ** = not done (sample not available)

### MOMP (cytochrome c assay)

**Figure 3:**
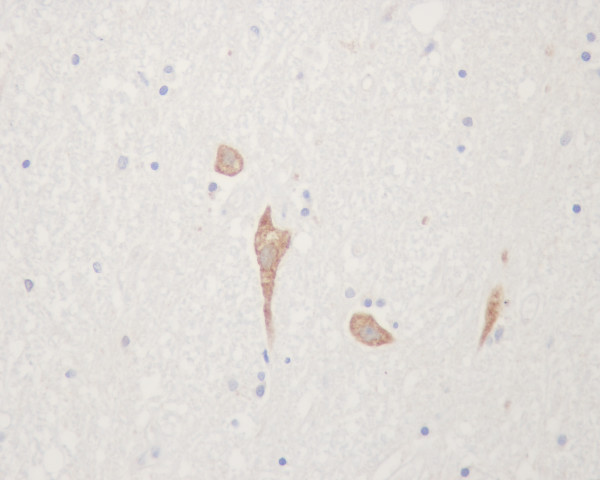
Cytochrome c positive neurons in brainstem at midbrain region of a paralytic rabies patient (no. H4 – see text and Table 1).

Evidence of MOMP was detected by demonstration of cytochrome c antigen in cytoplasm (Figure 3). In furious group, there was a discrepant result between the degree of rabies positive and cytochrome c positive neurons in spinal cord of patients with a survival period of 7 days or less (Patients no. 1, 5, 6) (Table 2). This was also noted in paralytic patient no. 4 who survived 7 days. Furious patient no. 8 did not have spinal cord specimens available. Of these 5, one of them (Patient no. 1) had brainstem (midbrain, pons, and medulla) negative for MOMP.

**Figure 4:**
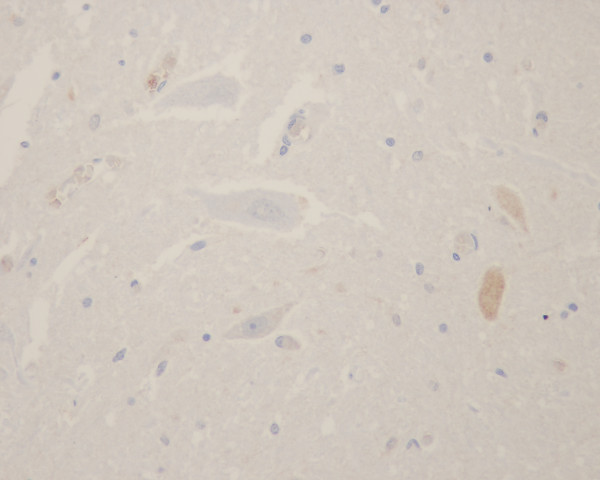
Cytochrome c positive neurons in spinal cord at thoracic region of a furious rabies patient (no. H3 – see text and Table 1).

Among 4 rabies patients who survived longer than 7 days with spinal cord specimens available, paralytic patient nos. 2 and 7 had relative absence of cytochrome c positive neurons as compared to rabies in all corresponding spinal cord regions (Table 3). Furious and paralytic patient nos. 3 and 10 had less degree of cytochrome c positive neurons in 3 out of 4 regions of spinal cord (Figure 4). Such discrepancy was noted in 2 or 3 regions of brainstem in 2 of 5 patients (paralytic patient nos. 9 and 2 respectively). Less degree of cytochrome c positive neurons was also evident in 1 of 3 regions of brainstem in paralytic patient nos. 7 and 10 (pons in one and midbrain in another).

### TUNEL assay

**Figure 5:**
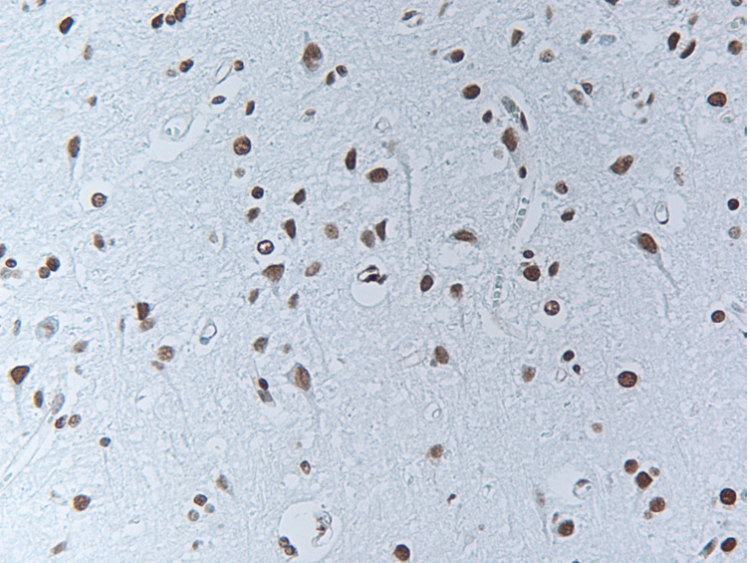
TUNEL staining. Apoptotic cells as demonstrated by TUNEL assay were found throughout the whole neuraxis in all patients.

Apoptotic cells as demonstrated by TUNEL assay were found throughout the whole neuraxis in all patients (Figure 5). There was no significant correlation between short or long survival period, amount of rabies antigen positive neurons and degree of apoptosis in various CNS regions (data not shown).

## Discussion

Our study showed that that some of neuronal cells especially in spinal cord and brain stem regions had a delay in apoptotic process especially that mediated via cytochrome c of the mitochondrial pathway. Although TUNEL assays did not reveal any differences among neurons at various regions, this was not surprising since varieties of unavoidable factors, such as hypoxia and ischemia, also contributed to apoptosis.

A relative absence of MOMP was found in spinal cord of rabies patients (8 of 8) regardless of clinical forms and survival period despite the presence of abundant amount of rabies virus antigen. Such phenomenon was also evident in one or more regions of brainstem in 5 of 10 patients (patient nos. 1, 2, 7, 9 and 10). Biting site did not correlate with either clinical forms or the abundance or absence of MOMP and rabies virus antigen.

Both *in vitro *and animal model observations in rabies agree to a similar conclusion that necrotic process is usually lacking with apoptosis becomes dominant findings [13,14]. The degree of apoptosis correlated with amount of expression of rabies G protein in infected neurons [15-17]. Nonfatal or abortive infection and the process of viral clearance is mediated by local recruitment of T cells, as well as the development of apoptosis of infecting neurons and surrounding cells [17]. Downregulation of G protein expression in neuronal cells contributes to pathogenesis by preventing apoptosis [18]. Although apoptosis may be a protective rather than a pathogenetic mechanism because less pathogenic viruses induced more apoptosis than more pathogenic viruses [16,19,20], all of our patients were bitten by street rabies virus variant transmitted by rabid dogs. Our previous study did not reveal any evidence of specific variants in association with the development of furious or paralytic presentation [3].

It remains intriguing why neurons, particularly those in spinal cord and brainstem are resistant to the effect of rabies infection. Previous *in vitro *model suggest this refers to an inherent property of spinal motoneurons themselves [21]. This may also be true in brainstem neurons. Furthermore, we found that amount of rabies virus in the brain should not be the sole contributing factor in determining the functional degree of brain functional alterations. Biopsy specimen of temporal lobe from a paralytic rabies patient who remained alert and rational showed large amount of rabies virus antigen on direct fluorescent test [22]. Magnetic resonance imaging showed abnormalities in the brain of a furious rabies patient who at that time did not exhibit any brain symptoms and signs [8]. He only had a local neuropathic pain at bitten left arm. Numerous studies point to the alterations at the levels of neurotransmitters, cytokines, ion channels, cellular RNA and protein synthesis and brain electroencephalographic patterns as well as role of neurotoxicity [7,22-38].

## Conclusion

In rabies virus infection, mechanisms involved in cell death or survival of neurons are complex [13,39,40]. Preservation of the neuronal network by inhibition of apoptosis and limitation of the inflammation and the destruction of T cells that invade the CNS is crucial for neuroinvasion [41]. This in addition to uncharacterized properties of certain neuronal cells may explain why spinal cord and brainstem where rabies virus was found heavily and early in the disease course, yet still retain their functions. We hope that by knowing what are unique among these types of neurons in term of response to rabies virus infection may give us ideas how to preserve neuronal functions and postpone death until native immunity (or any novel therapeutics) may arise for rabies virus clearance [38].

## Competing interests

The author(s) declare that they have no competing interests.

## Authors' contributions

SJ carried out laboratory work and histopathological examination, participated in data analysis and involved in drafting the manuscript. PR developed and optimized laboratory protocol and condition and involved in drafting the manuscript. SS participated in histopathological examination and data analysis and interpretation and involved in drafting the manuscript. SW participated in data analysis and interpretation and in drafting the manuscript. TH designed the study and coordination and involved in histopathological examination and data analysis and interpretation and writing the manuscript. All authors read and approved the final manuscript.

## Pre-publication history

The pre-publication history for this paper can be accessed here:


